# The Adoption of Genetically Modified Crops in Africa: the Public’s Current Perception, the Regulatory Obstacles, and Ethical Challenges

**DOI:** 10.1080/21645698.2024.2345401

**Published:** 2024-04-23

**Authors:** Gideon Sadikiel Mmbando

**Affiliations:** Department of Biology, College of Natural and Mathematical Sciences, University of Dodoma, Dodoma, Tanzania

**Keywords:** Cultural norms, ethical concern, farming practice, food security, GM crops, regulatory framework

## Abstract

Genetically modified (GM) crops are the most important agricultural commodities that can improve the yield of African smallholder farmers. The intricate circumstances surrounding the introduction of GM agriculture in Africa, however, underscore the importance of comprehending the moral conundrums, regulatory environments, and public sentiment that exist today. This review examines the current situation surrounding the use of GM crops in Africa, focusing on moral conundrums, regulatory frameworks, and public opinion. Only eleven of the fifty-four African countries currently cultivate GM crops due to the wide range of opinions resulting from the disparities in cultural, socioeconomic, and environmental factors. This review proposed that addressing public concerns, harmonizing regulations, and upholding ethical standards will improve the adoption of GM crops in Africa. This study offers ways to enhance the acceptability of GM crops for boosting nutrition and food security globally.

## 1. Introduction

One-third of the 800 million people who suffered from chronic malnutrition worldwide in 2017 were found in Africa.^1^ Despite having 25% of all arable land, 10% of global agricultural production originates in Africa.^2^ Low agricultural productivity can be attributed to several factors, including food loss and spoiling caused by pests and pathogenic microorganisms.^3,4^ Finding the best approach to increase agricultural yield in Africa is therefore critically important. Genetically modified (GM) crops produced by genetic engineering are one example of agricultural biotechnology that offers methods to improve food security and nutrition.^1,5,6^ The incorporation of GM crops into agricultural practices has gained global attention, with the impact of this integration being particularly noticeable in Africa’s diverse and agrarian landscapes.^5–7^ GM crops offers both potential and challenges,^6–9^ as nations struggle with the urgent need to safeguard food security and strengthen agricultural resilience.^10–13^ The public’s view of GM crops in Africa is characterized by a diverse range of beliefs and attitudes, which are shaped by rich cultural designs and varied socio-economic contexts.^14^ Comprehending these subtleties is essential for implementing public engagement and policy initiatives that work. Simultaneously, a variety of regulatory strategies used by African countries create a complex environment. These strategies range from strict prohibitions to cautious acceptance and affect both domestic agricultural practices and international cooperation.^5–7,15^ Furthermore, there are significant ethical concerns about the use of GM crops in African agriculture.^16,17^

Genetically modified organisms (GMOs), are living things, that include microorganisms, animals, or plants, whose genetic makeup has been intentionally changed in a laboratory setting through the use of biotechnological methods like genetic engineering.^18^ GM crops are created from plants that have undergone genetic alterations.^18,19^ For instance, Mmbando et al. recently developed a GM (transgenic) ultraviolet-B (UV-B)-resistant African rice cultivar (*Oryza glaberrima*) that has a raised UV-B resistance mechanism.^20^ When GM crops are strategically integrated, they can support growth in the economy, sustainable development, and resilience to changing environmental and socioeconomic conditions^1,6^ on a continent where a significant portion of the population relies on agriculture for a living.^21^ Comprehending public opinion, legal structures, and moral implications is crucial for the effective incorporation of GM crops in agricultural production. Adoption of GM crops is influenced by public acceptance, so it’s critical to resolve concerns and openly convey benefits.^22,23^ Strong regulatory frameworks prevent accidents during deployment, promote trust among parties, and enable cross-border cooperation.^15,24^ Responsible practices are guided by ethical considerations that help to mitigate potential negative effects on biodiversity, social equity, and conventional agriculture.^25,26^ However, the current regulatory frameworks are costly to local African institutions, ineffective with absence of transparency and extremely skeptical of risk.^1,27^ As there are currently insufficient policies, regulations, implementation, and monitoring/surveillance frameworks concerning GMOs, most African countries do not have regulations pertaining to the use of GM crops compare to the rest of the words.^1^

Previous studies about GMO adoption and regulation in Africa are focused on one aspect only, e.g., policy or regulatory rules or public perception.^1,5–7^ Comparing Africa to the United States of America (USA), Argentina, Canada, Brazil, and India, the total area under GM crop cultivation in Africa has remained notably low in 2017–2019.^6^ Adoption of GM crops has been extremely slow in African countries; only eleven of the fifty-four have current approved their cultivation (Table 1).^28,29^ Differentiated attitudes are influenced by a range of cultural, economic, and environmental factors.^30,31^ Furthermore, while earlier reviews^1,5–7^ discussed the regulatory obstacles African countries faced when adopting GM crops, there is still a knowledge vacuum regarding the precise effects of these regulatory roadblocks on the rate and scope of GM crops adoption. Therefore, it is crucial to identify the complex factors that prevent GM crops from being widely adopted in Africa. There is, however, a dearth of recent data regarding this issue. This study integrates the public’s current perception of GMOs, the intricate regulatory obstacles, and the ethical dilemmas at play to provide a comprehensive view. By doing this, it provides an in-depth understanding of the benefits and difficulties associated with the adoption of GMOs in Africa, which is essential for creating effective plans and regulations. This comprehensive approach will enables stakeholders and policymakers to address issues from multiple angles, which facilitates better decision-making and may speed the responsible integration of GM crops into African agriculture.

**Table 1.** The current African nations with approval of genetically modified (GM) crops. Only eleven of the fifty-four African nations—Burkina Faso, Egypt, eSwatini, Malawi, Ethiopia, Ghana, Kenya, Nigeria, South Africa, Sudan, and Zambia – as of this moment have approved the cultivation of GM crops. Source^28,29^.

SN	Country	Crop	Scientific name	Events approved	Biosafety laws and GM commercial crops
1	Burkina Faso	Cotton	*Gossypium hirsutum* L.	1	YES
2	Egypt	Maize	*Zea mays* L.	1	YES
3	eSwatini	Cotton	*Gossypium hirsutum* L.	2	YES
4	Ethiopia	Cotton	*Gossypium hirsutum* L.	2	YES
5	Ghana	Cowpea	*Vigna unguiculata*	1	YES
6	Kenya	Cotton	*Gossypium hirsutum* L.	2	YES
		Maize	*Zea mays* L.		YES
7	Nigeria	Cotton	*Gossypium hirsutum* L.	29	YES
		Cowpea	*Vigna unguiculata*		YES
		Maize	*Zea mays* L.		YES
		Soybean	*Glycine max* L.		YES
		Wheat	*Triticum aestivum*		YES
8	South Africa	Argentine Canola	*Brassica napus*	75	YES
		Cotton	*Gossypium hirsutum* L.		YES
		Maize	*Zea mays* L.		YES
		Rice	*Oryza sativa* L.		YES
		Soybean	*Glycine max* L.		YES
9	Sudan	Cotton	*Gossypium hirsutum* L.	1	YES
10	Zambia	Maize	*Zea mays* L.	6	NO
11	Malawi	-	-	-	YES

**Figure 1.**
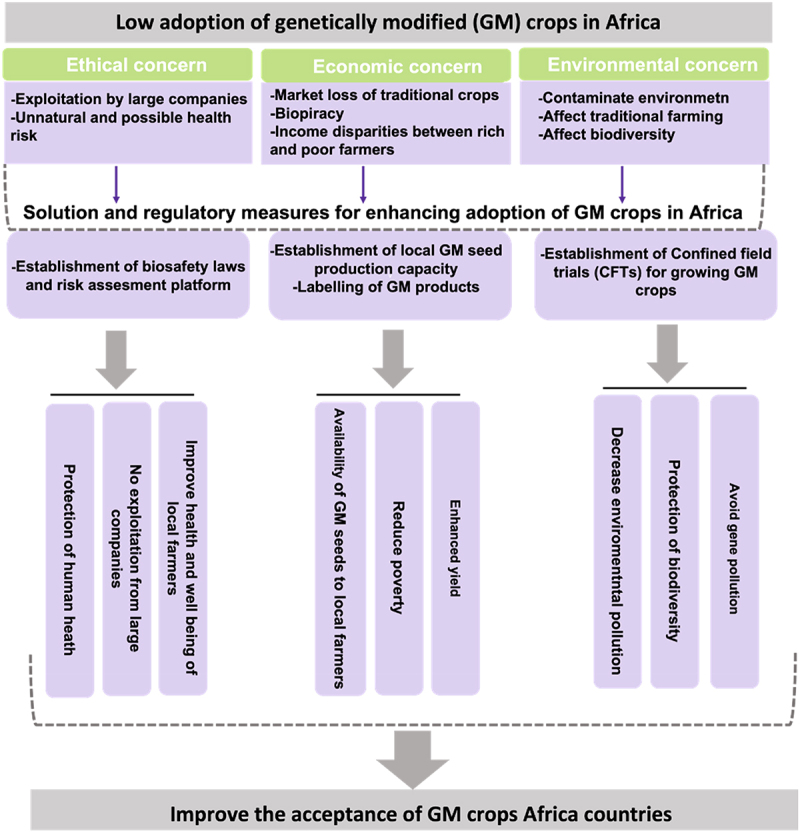
Possible solutions for factors affecting the acceptability of genetically modified (GM) crops in Africa.

This review provided light on the current state of adopting GM crops in Africa by offering a thorough grasp of the opportunities and difficulties that arise from the convergence of ethical concerns, legal frameworks, and public opinion. With an emphasis on the dynamic interactions between public perception, legal frameworks, and ethical concerns, this review will disentangle the complex web of factors surrounding the use of GM crops on the African continent (Fig. 1). This review’s investigation will help make well-informed decisions, supporting a long-term, inclusive strategy for integrating GM crops into African agriculture.

Graphic representation of the factors for countering the rejection of GM crops in the majority of countries in Africa. The establishment of a combination approach, which includes biosafety laws and risk assessment procedures, local GM seed production facilities, and confined field trials (CFTs) for commercialization, will reduce worries and build trust and confidence in GM products. This will improve the adoption of GM crops in many African countries.

## 2. The Current Situation Regarding the Use of GM Crops in Africa

Africa’s agriculture is incredibly important and is considered the continent’s lifeline. It is the foundation of many economies, providing a significant percentage of the population with a means of subsistence.^32^ Agriculture offers food and revenues in rural areas where subsistence farming is common, promoting a stable economy.^32,33^ Apart from food production, the sector provides raw materials for other industries and jobs in both the official and unofficial sectors.^34,35^ Numerous agricultural pursuits, ranging from the production of staple crops to the rearing of livestock, are made possible by Africa’s varied climates and environments. Furthermore, agriculture is essential to solving urgent problems like rural growth, food security, and poverty reduction.^36,37^ Understanding and promoting the importance of agriculture is crucial for promoting sustainable development and resilience in the face of shifting socio-economic and environmental dynamics as the continent deals with the difficulties of modernization and global markets.

The adoption of GM crops has progressed from early promise to a contentious environment.^1,6–8,38,39^ GM crops were first heralded as a technological blessing, offering ground-breaking answers to the world’s most pressing agricultural problems. Their introduction offered a promising solution to food security issues because of their potential to boost crop yields, boost nutritional profiles, and strengthen resilience to pests and diseases.^18,40,41^ This hopeful path has, however, been greeted with an increasing number of disputes. For example, worries about unintended impacts on non-GM crops, long-term adverse environmental impacts, and corporate consolidation of agricultural control have increased the public’s fear^42–44^ The safeguarding of conventional agricultural methods, the growing number of multinational corporations, and socioeconomic disparities have all raised ethical concerns.^42,44^ The growth trajectory of GM crops is a diverse story that emphasizes the importance of having a sophisticated comprehension of the ethical, environmental, and socioeconomic factors involved.^17,39^ It emphasizes how crucial it is to have open lines of communication, strong legal protections, and thorough risk analyzes to successfully negotiate the changing GM crops debate.

An intricate narrative surrounding the acceptance of GM crops in Africa has been shaped by worries about the ecosystem, unforeseen outcomes, and moral implications. A sophisticated grasp of socioeconomic dynamics, open communication, and flexible regulatory frameworks are necessary to navigate this evolution.^24,45,46^ The adoption of GM crops in Africa has followed a trajectory that strikes a balance between the benefits and drawbacks, mirroring the larger global conversation about using biotechnology to promote fair and sustainable farming methods.^1^ GM crops acceptance or distrust is shaped by current public perception, which is strongly rooted in cultural, economic, and environmental factors.^6,22,23,43^ It is imperative to comprehend and tackle a range of concerns to promote well-informed dialogue and acceptance. Furthermore, regulatory frameworks in Africa vary widely in their approaches, ranging from cautious approval to outright prohibitions.^5,24^ To support research projects, regional collaboration, and the safe application of GMOs, these frameworks must be harmonized.^15,45^ Moreover, social justice, environmental consciousness, and cultural values are all entwined with ethical issues. However, It is a challenging task to strike a balance between the potential for increased agricultural productivity and moral worries about conventional farming methods, economic inequality, and biodiversity preservation. This tripartite investigation highlights the necessity for comprehensive strategies as Africa negotiates the incorporation of GM crops into its agricultural terrain. Realizing the prospective benefits of GM crops while guaranteeing ethical and environmentally friendly agricultural practices on the continent will require bridging knowledge gaps, harmonizing guidelines, and navigating ethical complexities.^15,22,47^ The future course of the implementation of GM crops in Africa will be significantly influenced by a thorough examination of these factors.

## 2. Current Public Opinions Regarding GM Crops Use in Africa

A complex interplay of customs, beliefs, and social norms that influence perceptions is revealed by the investigation of cultural factors determining people’s views toward GM crops in Africa.^5,22^ Numerous countries with distinct cultures throughout the continent have distinct attitudes toward agriculture and frequently see it as essential to their identity. For instance, GM crops may be viewed with doubts in certain communities where agricultural practices are strongly embedded in their cultural heritage because of worries about potential alterations to their traditional methods.^6^ Concerns regarding the dignity of nature and biodiversity, as well as spiritual beliefs and ties to the land, also affect how people perceive GM crops.^43,48^ Views regarding the ownership and utilization of GM seeds are influenced by the deeply ingrained concept of “biopiracy” in certain African cultures, which refers to the profit-driven use of naturally occurring genetic materials or biochemicals.^1,49^ Effective interaction and participation strategies require an understanding of these cultural nuances. A more sophisticated and culturally sensitive approach to the adoption of GM crops can be achieved through initiatives that include community leaders, appreciation and encompass local values, and encourage a collaborative dialogue between scientists and communities.^16,17,50^ This investigation emphasizes how important it is to acknowledge and manage cultural differences as essential elements of the larger discourse on GM crops in Africa.

The perception of GM crops in Africa is largely shaped by socioeconomic factors, which range from considering them as a way to address issues related to food security to seeing them as a danger to traditional livelihoods.^46,51,52^ GM crops frequently show promise as a means of reducing hunger, boosting crop resilience, and enhancing agricultural productivity in areas that struggle with ongoing food insecurity. For instance, some see economic opportunities in promoting the use of GM crops, with a possible rise in income and yields.^6,51^ On the other hand, some individuals express concern about possible disturbances to current farming practices, the disappearance of native agricultural expertise, and the financial imbalances resulting from the commercialization of seeds.^9,17^ Skepticism is influenced, for instance, by worries about gene flow to wild plant populations, possible effects on organisms other than the intended target, and uncertainties about long-term ecological consequences.^1,6,16^ These worries are heightened by the multitude of ecosystems found in Africa, where unique climates and biodiversity hotspots raise worries about how well-suited GM crops are to a range of environments.^53,54^ Thus, the perceived advantages and hazards related to the environmental effects of GM crops have a consequence on public perceptions.^22,55^ A careful balance must be struck when navigating these socioeconomic viewpoints to protect the socio-cultural fabric of communities that rely upon traditional farming methods while guaranteeing the advantages of GM crops contributing to wider economic development.

Due to the complex communication issues regarding the use of GM crops in Africa, tactical strategies are needed to allay public fears and misconceptions.^15,17,56^ Culture, socioeconomic status, and educational attainment all have a role in people’s perceptions of GM crops. This leads to a variety of perspectives and the need for customized communication tactics.^50,56^ Clear and easy access to information outlining the possible advantages and disadvantages of GM crops is essential for effective engagement. Initiatives promoting science literacy and understanding are essential because misinformation can lead to misconceptions.^6,22,57^ Culturally appropriate communication that respects local knowledge and considers community perspectives can help close this knowledge gap. Building trust is also necessary for decision-makers, scientists, and the general public to communicate effectively with each other.^9,58^ It is critical to address communication barriers to create a well-informed public dialogue that takes into account a range of viewpoints and encourages a fair assessment of the contribution of GM crops to the solution of Africa’s agricultural problems.

## 3. Regulations and Policies About the Use of GM Crops Throughout the African Continent

Analysis of the regulatory environment surrounding GM crops in several African nations demonstrates a wide range of perspectives and beliefs regarding agricultural biotechnology.^6,7,24^ Although some countries have welcomed GM crops as a way to improve productivity in farming and food security, others have put cautious regulatory frameworks in place that emphasize careful safety evaluations and risk management. On the other hand, some African nations continue to impose strict laws or complete prohibitions due to worries about possible negative effects on the environment, human health, and socioeconomic conditions.^1,5–7^ For example, South Africa has developed a broad regulatory framework that permits the commercial production of specific GM crops, demonstrating a comparatively positive position.^9,59^ It is also known that although some nations, like Egypt and Burkina Faso, were among the first to use GM crops in Africa, there are now back slided. Furthermore, Ethiopia, Kenya, Ghana, Nigeria, and eSwatini are among the countries that are just beginning to use this technology in their agricultural systems. Conversely, nations such as Tanzania, Uganda, and Zimbabwe have implemented more stringent policies, prohibiting the growing of particular GM crops (Table 1).^7,28,29,60^ GM crops and technological advancements are only useful for research under carefully monitored circumstances. Research efforts, trade, and regional collaboration are hampered by the regulatory frameworks’ absence of harmonization.^7,15^ To close these gaps, policymakers must have a thorough awareness of the particular conditions of every country and engage in continuous discussion to create regulations that strike a balance between the possible advantages of GM crops and safety and environmental concerns.

GM crops regulation takes many different forms throughout the African continent: acceptance, cautious application, and outright prohibition.^7,15,60^ This compromise strategy addresses the need for food security while taking socioeconomic and environmental factors into account. It aims to strike a balance between potential advantages and public worries.^22,42^ The absence of harmonization makes it more difficult for resources and knowledge to flow freely, which hinders the creation of long-term, locally appropriate solutions to agricultural problems. Different laws and regulations make it difficult for research programs to conduct cross-border studies, which results in dispersed efforts and retarded discoveries in science.^7,15^ Farmers face uncertainty when it comes to implementing GM crops in agriculture because of the uneven regulations that hinder the uniform application of GMOs.^39,61^ Although they are from the same regions, East African nations like Kenya, Tanzania, and Uganda, for instance, have varying laws governing the use of GM crops. At the moment, Tanzania and Uganda oppose the use of GM crops for improving agricultural yields while Kenya does.^7^ This makes it more difficult to improve agricultural productivity and solve issues related to food security at the continental level. Harmonized regulatory structures that take into account the various contexts of the continent’s agricultural landscapes are desperately needed to realize the full benefits of multinational collaborations, promote significant research, and guarantee the safe acceptance of GM crops in Africa.^15^ Harmonization will play a pivotal role in advancing sustainable development and tackling the distinct obstacles encountered by African countries.

Emerging patterns in GM crops regulatory strategies suggest that certain nations are investigating flexible regulatory frameworks that strike a balance between promoting agricultural innovation and safety concerns.^6,24,60^ This calls for a more flexible strategy that takes into account risk assessments and continuous scientific developments to guide regulatory choices.^8,22^ Furthermore, cooperative initiatives are gaining momentum as regional blocs seek to harmonize GM crops regulations.^9^ Process simplification, international cooperation, and a more unified approach to GM crops governance are the goals of this movement. Another developing trend is public participation, which aims to involve a variety of stakeholders in the process of decision-making.^47^ The goals of inclusive approaches are to resolve issues, improve openness, and increase public confidence in regulatory frameworks.

National policies and regulatory frameworks are frequently shaped by the views of political leaders regarding GM technology.^22,50,62^ The adoption of GM crops is generally supported by governments that prioritize agricultural modernization and food security, with the goal of increasing yields and addressing agricultural issues.^63^ On the other hand, the government impacted by anti-GMO beliefs might implement limitations, apprehensive about possible hazards or negative public reaction toward GM crops.^7,62^ For instance, among the nations of East Africa, Tanzania and Uganda do not support the use of GM crops, whereas Kenya’s government does.^7^ The decisions made about GM agriculture can be influenced by international politics, trade agreements, and foreign relations.^39^ While some countries may oppose the adoption of GMOs in order to safeguard their own markets or customary farming methods, others may seek partnerships with countries that produce GMOs by aligning their policies with partner standards to facilitate trade.^6,64^ Moreover, effective adoption of GM crops depends on domestic political stability.^62,65^ While insecure political environments can cause policy uncertainty and delays, transparent regulations and institutional capacity guarantee seamless implementation.

Effective biosafety laws offer a methodical way to evaluate, control, and keep an eye on possible risks related to GMOs.^5,24^ A strong biosafety framework encourages the responsible use of GMOs by fostering confidence among stakeholders, such as farmers, consumers, and policymakers. For instance, every county that permitted the cultivation of GM crops had a functional biosafety framework, with Zambia being the exception (Table 1).^28,29^ Therefore, a biosafety framework needs to be established in most African countries in order to increase the adoption of GM crops.

## 4. The Ethical Challenges Affecting Africa’s Present Use of GM Crops

A careful analysis of values, equity, and sustainability is involved in the investigation of ethical issues related to the use of GM crops in Africa.^22,66,67^ Potential power concentration in the agriculture industry is a major ethical concern, as large corporations control large portions of the seed market and agricultural procedures.^1,64,68^ Furthermore, the effect on indigenous knowledge systems and customary farming methods presents moral dilemmas for the preservation of cultural heritage.^16,69,70^ Concerns also include social justice issues, such as preventing economic inequality and providing small-scale farmers with fair access to GMO technologies.^71,72^ According to Article 26 of the Cartagena Protocol on Biosafety, parties may take into account socioeconomic factors that may arise from the import of living modified organisms (LMOs) and have an impact on biodiversity conservation and sustainable utilization, particularly in light of the importance of biological diversity to local and indigenous groups.^73^ Unfortunately, most African nations such as Kenya lack the necessary information for analysis, evaluation, and inclusion in biosafety decision-making.^46,66,74^ Thus, it is possible that most developing poor African countries failed to look the social-economic implications of LMOs’ effects on biological diversity. This might have been a significant barrier to the adoption of GM crops in many African countries, leading them to favor the precautionary principal.

Moreover, the 2010 Nagoya Protocol, an international framework created in 2010, placed a strong emphasis on guaranteeing access to genetic resources as well as ensuring an equal distribution of the advantages that result from their use.^75,76^ Even though 48 African nations had already ratified the Nagoya Protocol as of July 2022,^77^ the effectiveness of the Nagoya Protocol’s guiding principles may be hampered by regional variations in national policies and practices.^75^ For instance, there are differences in the degree of involvement with the Access and Benefit-Sharing (ABS) Clearing House among the regions of the Southern African Development Community (SADC). Indeed, Ivey et al., have proposed that a number of African nations do not currently have protocols and guidelines in place to carry out the Nagoya Protocol and manage access to possible biological control agents.^78^ Similar to biological agents, delays in countries implementing ABS for GM crops may result from a lack of knowledge, inadequate capacity, and relevant information, which will lower the adoption and acceptability of GM crops. Research, managers, and bureaucrats must thus work together to support African nations, as this could result in a collective effort that creates policies and puts procedures in place to encourage the investigation of the potential benefits of GM agriculture. Through this partnership, resources, technology, and GM crop seeds could be shared, thereby improving food security throughout Africa. It becomes essential to weigh the possible advantages of GM crops in resolving issues with food security against these moral considerations. To ensure that the incorporation of GM crops is compliant with ethical principles and promotes sustainable and fair agricultural development, an ethical framework must place a high priority on inclusive decision-making processes, transparency, and concerns for the distinct cultural and socioeconomic situations within Africa.^42,72^

The adoption of GM crops may cause established agricultural norms to change, upending long-standing traditional farming practices that are deeply rooted in the history of the continent.^69,70,79^ The diverse range of traditional farming methods may be in danger due to a possible shift toward monoculture and commercialized agriculture.^5,9^ Furthermore, traditional systems of knowledge that have supported crop cultivation for many generations may be threatened by GM crops. The adoption of biotechnological methods has the potential to challenge conventional wisdom and affect how agricultural knowledge is passed down through communities.^16,70^ Heritage may be threatened by this, which could result in the extinction of unique farming techniques and the disintegration of cultural identities that have their roots in agriculture.^80^ To effectively navigate these effects, ethical considerations must be given careful thought, inclusive decision-making must be implemented, and modern agricultural methods must be integrated with methods for preserving traditional knowledge and cultural heritage.

Furthermore, adopting GMO technology and buying GM seeds can lead to disparities in cost that favor larger, wealthier farming entities.^8,46,81^ For this reason, economic equity becomes a central concern. The possibility of a greater reliance on outside sources for technology and seeds may intensify economic disparities in the agriculture industry.^64,68^ The equitable distribution of the advantages of GM crops adoption across a range of agricultural landscapes is based upon the careful evaluation of inclusive policies, equitable access to resources, and mechanisms that empower small-scale farmers. There is an urgent need for a sensible strategy that upholds moral standards and maximizes the possible advantages of GM crops in Africa. Cultural norms, environmental sustainability, and social justice are all included in the ethical framework. It takes a nuanced and inclusive viewpoint to balance these against the possible benefits of GM crops, such as higher crop yields, pest resistance, and improved nutritional content.^6,42,46^ An equitable strategy recognizes the varied socioeconomic backgrounds of the continent and addresses public concerns through open communication is needed. Policies must be put in place that give safety and morality top priority while allowing for technological advancement. Furthermore, incorporating indigenous knowledge systems and guaranteeing local communities’ involvement in decision-making processes promotes a more moral and sustainable incorporation of GM crops into African agriculture.^72^ Through careful consideration of these subtleties, a well-rounded strategy can help resolve issues related to food security while respecting moral principles and protecting the continent’s varied natural and cultural environments.

## 5. Case Studies and the Effective Implementation of GM Crops in Certain African Nations

GM crops adoption in agriculture has proven successful in some African nations, demonstrating favorable effects on resilience, productivity, and sustainability.^1,6,15^ One noteworthy example of success is in South Africa, where commercial production of *Bacillus thuringiensis* (*Bt*) Zea mays L. and *Gossypium hirsutum* L., which are resistant to insects, has greatly raised yields and decreased the requirement for chemical pesticides.^16,82,83^ Farmers’ incomes have increased as a result of the adoption of these GM crops, indicating that there are potential economic advantages.^46,71^ Furthermore, Burkina Faso has seen significant advancements as a result of the adoption of *Bt G. hirsutum* L. Although the country is now is back slide, farmers saw gains in income, lower dependency on chemical pesticides, and higher yields. The progress made in South Africa shows that, despite certain disagreements,^84^ GM crops have the potential to support sustainable farming methods and economic advancement.^46,85^ Another notable example is the growth of GM pod-borer-resistant *Vigna unguiculata* (known as SAMPEA 20-T), which was authorized and widely accepted in Nigeria in 2019. The *Bt V. unguiculata* demonstrates resilience against the pod borer, a destructive pest.^86^ Higher yields and less pesticide use are reported by farmers growing *Bt V. unguiculata*, which improves food security.^87^

In addition, Kenya declared at the close of 2019 that commercialization of *Bt G. hirsutum* L. has begun, with plantings scheduled to begin in 2020.^88^ This happened after gene-editing crops were prohibited for seven years in Kenya.^1^ Kenyan farmers, however, have since revised their outlook and now hope for a smooth switch to *Bt G. hirsutum* L. in 2020.^88^ These achievements highlight how GM crops may be used to help African countries with particular agricultural issues. Along with higher yields and financial gains, the use of GM crops has reduced dependency on chemical inputs, improving environmental sustainability.^16^ But to truly appreciate these achievements, one must have a thorough awareness of the various contexts in which GM crops are embraced. The acceptance of GM crops is significantly influenced by various local factors, such as regulatory frameworks, socioeconomic circumstances, and cultural beliefs. African nations can effectively address fears and promote sustainable agricultural practices while exploring the expected benefits of GM crops by taking into account the distinct conditions of each region and drawing lessons from these accomplishments. Other African nations like eSwatini, Malawi, Ethiopia, and Sudan have also fully commercialized GM *G. hirsutum* L. (Table 1). Farmers in those nations have already profited from these modified crops, despite opposition of GMOs from neighboring countries.^81,89,90^

African cases show how public attitudes and the results of using GMOs can be positively impacted by ethical concerns, inclusive regulatory frameworks, and efficient communication. For example, open communication about the advantages of the crop allowed for the successful cultivation of *Bt G. hirsutum* L. in Kenya.^8,22,91^ Farmers, stakeholders, and the general public were engaged to debunk myths and foster confidence in the technology. Furthermore, Ethiopia included a range of stakeholders in the decision-making process concerning the adoption of GMOs.^9,92^ Due to the participatory model’s ability to take into account a variety of viewpoints, more thorough regulatory frameworks were produced. This method helped to further educate the public while also addressing ethical issues.^93,94^ Moreover, the effective implementation of *Bt Zea mays* L. and *G. hirsutum* L. in South Africa demonstrates the advantages of a balanced regulatory strategy. The nation has successfully handled the coexistence of GM and non-GM crops, allaying concerns and proving that biotechnology is compatible with a range of farming practices.^16,45^ These incidents highlight how crucial open communication, inclusive governance, and moral considerations are to fostering the adoption of GM crops in Africa. Establishing public trust and promoting the responsible use of GM crops in agriculture can be achieved through a comprehensive strategy that incorporates a range of stakeholders, honors cultural values, and places a high priority on safety and sustainability.

Furthermore, another major obstacle for implementation of GM crops in most African countries is the absence of well-established seed systems and infrastructure for the production and distribution of GM crops.^68,91^ This is due to the fact that the development and propagation of GM seeds necessitate specific expertise and resources, both of which may be lacking in many developing African nations. For instance, the *Bt. G. hirsutum* L. seeds used in Kenya, Sudan, Ethiopia, Nigeria, and e-Swatini are imported rather than produced domestically in those nations, which drives up the cost of the seeds for farmers.^28,84^ As a result, smallholder farmers adopted GM crops and had limited access to GM seeds. Therefore, Africa needs to establish and improve its local seed production facilities and capacity in order to increase the adoption of GM crops. Moreover, public-private partnerships can also be very helpful in increasing the accessibility and availability of GM seeds.^63^ This will eventually help GM crops become widely adopted and improve food, nutrition, and health outcomes in African nations. For instance, in Uganda, HarvestPlus and other organizations have been instrumental in providing farmers with access to vitamin A-rich orange-fleshed sweet potatoes (OFSP) through partnerships with agricultural extension services and seed distribution programs.^95,96^ Thus, governments, non-governmental organizations, and partners in the private sector must work together to coordinate efforts in order to address the infrastructure and distribution challenges.

## 6. Prospects and Suggestions for the Future

The adoption of GM crops in Africa may be trending in a different direction due to changing agricultural requirements and technologies. Growing knowledge and comprehension of the advantages of GM crops could result in increased acceptance, as more nations investigate biotechnological solutions to food security issues.^7,9,15,17,51,60^ Though little is known in Africa, cooperative efforts to standardize regulatory frameworks could expedite procedures and enable cross-border adoption of GM crops in a responsible manner.^7,15,39^ Moreover, the creation of crops with higher nutritional content, resistance to drought, or tolerance to diseases could spur interest in GM crops due to genetic engineering improvements.^42,97^ A more focused and palatable approach could be provided by the use of precision biotechnologies, such as gene editing e.g clustered regularly interspaced short palindromic repeats (CRISPR)-associated endonuclease Cas9 (CRISPR/Cas9), which could impact the direction of GM crops approval in African agriculture.^64,98,99^ However, ongoing discussions about socioeconomic, environmental, and ethical issues will probably affect these trends.^22,42,44,46,67^ The way that GM crops adoption plays out in Africa in the future will be determined by how well the possible advantages are weighed against ethical and environmental considerations.

Addressing public concerns about GM crops should be a top priority for African stakeholders, researchers, and policymakers through open and honest communication. Building trust and fostering understanding can be accomplished through inclusive dialogs involving a variety of stakeholders.^15^ Establishing and harmonizing regulatory frameworks that strike a balance between safety concerns and the potential advantages of GM crops is essential to support international research partnerships and initiatives. In addition, social justice, economic equity, and the safeguarding of environmental and cultural values must be given top priority to uphold ethical principles.^24^ Lawmakers ought to place a strong emphasis on creating regulations that are tailored to the unique circumstances and values of the local community. Prioritizing research should go toward evaluating how GM crops affect ecosystems and conventional farming methods over the long run.^100,101^

Working together, stakeholders – farmers, scientists, and legislators – can guarantee that the adoption of GM crops respects ethical principles, is consistent with sustainable development objectives, and responsibly responds to public concerns.^5,6,9^ For responsible GM crops use to be supported in the African context, international collaboration is essential.^39^ Working together makes it easier to share knowledge, which guarantees that African countries profit from biotechnology breakthroughs worldwide while taking into account their particular agricultural difficulties. The exchange of efficient procedures in risk assessments, regulatory frameworks, and public engagement will help African policymakers make better decisions. International cooperation will also encourage consistency, lower trade barriers, and harmonize regulatory standards. This will facilitate collaborative research projects aimed at addressing regional needs, like cultivating crops resistant to regional pests or climate variations. Furthermore, by combining various viewpoints and values into GM crops adoption plans, collaboration promotes ethical considerations. In the end, international collaboration fosters an atmosphere that is favorable for the responsible use of GM crops in Africa, enabling countries to manage the challenges of agricultural biotechnology while preserving social, economic, and environmental interests.

## 7. Conclusion

The review clarifies the recent complex dynamics of GM crops adoption in Africa by highlighting the interplay between public perception, regulatory obstacles, and ethical considerations. Diverse public opinions throughout the continent highlight the necessity of context-specific strategies that take into account subtleties in culture, socioeconomic status, and the environment.^1,6,7,30,44^ Regulations illustrate the difficult terrain that policymakers must traverse, from acceptance to cautious implementation to outright prohibitions. The discourse is further complicated by ethical quandaries, which call for a careful balancing act between technological innovation and socio-cultural values. A sophisticated understanding of regional disparities becomes essential as Africa struggles with issues related to food security and sustainable agricultural practices. Dealing with the complexity of GM crops use in Africa requires a comprehensive and inclusive strategy. This entails ethical considerations for environmentally friendly farming, coherent regulations, and open communication. The adoption of gene-editing laws in African nations will accelerate the precise and rapid application of methods like CRISPR/Cas9, increasing the nutritional content and productivity of the continent’s agricultural sector. In the future, it will be crucial to promote open communication, inclusive decision-making procedures, and cooperative global initiatives. Responsible GM crops adoption will be made possible by tackling public concerns, harmonizing regulations, and upholding ethical standards. This will guarantee that technological advancements are in line with Africa’s diverse landscapes while contributing to a future of agriculture that is both equitable and sustainable. This study offers policymakers and stakeholders vital information that can improve the acceptance of GM crops and raise global food security.

## Data Availability

All data produced during this study are incorporated in the manuscript.
